# Urology-Related Conditions Should Not Be Overlooked in Women with Polycystic Ovary Syndrome

**DOI:** 10.1055/s-0041-1736552

**Published:** 2021-11-16

**Authors:** Tariq Faisal Al-Shaiji

**Affiliations:** 1 Urology Unit, Department of Surgery, Jaber Al-Ahmad Al-Jaber Al-Sabah Hospital, Kuwait City, Kuwait

Dear Editor,

It has been documented that women with polycystic ovary syndrome (PCOS) may suffer from sequelae that can surpass the obstetrics and gynecology armamentarium. For example, Shah and Rasool 1 gathered data from the literature showing clear evidence that PCOS is associated with an increased risk of developing type-2 diabetes mellitus, hypertension, dyslipidemia, endometrial cancer, and subclinical atherosclerosis. Therefore, screening for these disorders at the initial contact with these patients, as well as periodically, is recommended.

The findings reflecting the extent of the PCOS sequelae are intriguing. Nevertheless, I would like to highlight some points. We should look further beyond when it comes to PCOS and other associated diseases. The syndrome has been specifically implicated in a few urological conditions as well. In 1988, Fowler et al. 2 first described Fowler syndrome in young women with unexplained complete urinary retention with a unique abnormal pattern of electromyographic (EMG) activity and clinical features of PCOS. They suggested that the abnormal EMG activity was driven by a relative deficiency of progesterone leading to impairment of the urinary sphincter relaxation. 2 Meanwhile, other authors 3 have speculated that hyperoestrogenemia in PCOS might impair the relaxation of the urethral sphincter, leading to urinary retention. It is believed that PCOS in women with Fowler syndrome occurs in ∼ 64% of the cases. 4 Furthermore, It has been hypothesized that PCOS may lead to urolithiasis by triggering stone formation in the urinary tract. 5

Therefore, given that PCOS and these urological issues seem to be correlated, it is advised that women treated for one condition be screened for the other condition and vice versa. I hope that the aforementioned facts can add to the general awareness for women with PCOS and widen the circle of clinical assessment for these patients.
